# Improving postharvest quality and vase life of cut rose flowers by pre-harvest foliar co-applications of γ-aminobutyric acid and calcium chloride

**DOI:** 10.1038/s41598-024-64021-8

**Published:** 2024-06-24

**Authors:** Narges Ehsanimehr, Mehdi Hosseinifarahi, Moslem Abdipour, Saeid Eshghi, Babak Jamali

**Affiliations:** 1 Department of Horticultural Science, Yasuj Branch, Islamic Azad University, Yasuj, Iran; 2 Sustainable Agriculture and Food Security Research Group, Yasuj Branch, Islamic Azad University, Yasuj, Iran; 3 Kohgiluyeh and Boyerahmad Agricultural and Natural Resources Research and Education Center, Agricultural Research, Education and Extension Organization (AREEO), Yasuj, Iran; 4 Department of Horticultural Science, School of Agriculture, Shiraz University, Shiraz, Iran; 5 Department of Agriculture, Minab Higher Education Center, University of Hormozgan, Bandar Abbas, Iran

**Keywords:** γ-aminobutyric acid, *Rosa hybrida*, Postharvest, CaCl_2_, Plant cell biology, Plant physiology

## Abstract

Rose flowers (*Rosa hybrida* L.) are highly perishable and have a limited vase life. This study evaluated the effects of preharvest foliar applications of γ-aminobutyric acid (GABA) and calcium chloride (CaCl_2_), individually and combined, on antioxidant responses and vase life of cut Jumilia rose flowers. Treatments included foliar sprays of GABA at 0, 20, 40, and 60 mM and CaCl_2_ at 0, 0.75%, and 1.5%, applied in a factorial design within a completely randomized setup before harvest. Results showed GABA and CaCl_2_ interaction (especially, 60 mM GABA and 1.5% CaCl_2_) significantly increased enzymatic antioxidants including superoxide dismutase, catalase, and peroxidase, as well as non-enzymatic antioxidants such as flavonoids, carotenoids, phenolics, and antioxidant activity in petals compared to control. SOD activity in roses, treated with CaCl_2_ (1.5%) and GABA (60 mM), peaked at 7.86 units. mg^−1^ protein min^−1^, showing a nearly 2.93-fold increase over the control (2.68 units. mg^−1^ protein min^−1^). A parallel trend was observed for CAT activity. These treatments also reduced petal malondialdehyde content and polyphenol oxidase activity. Protein content and vase life duration increased in all treatments. Plants treated with a combination of GABA (20 mM) and CaCl_2_ (0.75%), GABA (60 mM) and CaCl_2_ (1.5%), or GABA (40 mM) individually exhibited the longest vase life duration. The co-application of GABA and CaCl_2_ improved the antioxidant activity and postharvest quality of cut roses by reducing PPO activity and MDA contents, increasing protein content and prolonging vase life. This treatment is a potential postharvest strategy to improve antioxidant capacity and delay senescence in cut roses.

## Introduction

Roses (*Rosa hybrida* L.) are considered the most diverse and widespread commercial flower in the global ornamental plant industry^1^. In terms of production and economic importance, they rank first due to their growth pattern, wide range of flower colors, and variety of flower shapes^2^. When it comes to the marketing appeal of roses, a number of characteristics play a crucial role in meeting consumer demands and enhancing the value and significance of roses in the marketplace. These include factors such as flower color, stem diameter/length, flower bud height/diameter, and, most importantly, vase life^3^. This vital parameter, i.e., vase life, depends on a range of factors, e.g., variety, optimal growing conditions, a/biotic stresses, harvesting processes, post-harvest handling procedures, etc.^4^. Fertilization management and some plant growth bioregulators application during plant growth improve the quality and vase life of cut flowers.

γ-aminobutyric acid (GABA) is a four-carbon, non-proteinogenic amino acid. This naturally occurring bioactive compound is crucial in various plant physiological processes, such as growth, signal transmission, and stress responses. Research has demonstrated that applying GABA before and after harvest can prolong the vase life of numerous cut flowers. These include rose^5^, gerbera^6,7^, carnation^8^, protea^9^, anthurium^10,11^, daffodil^12^, and tuberose^13^. The mechanisms behind this extension of flower vase life are complex. GABA appears to regulate the expression of genes involved in hormone biosynthesis, transcriptional regulation, reactive oxygen species (ROS) generation, polyamine metabolism, and both enzymatic and non-enzymatic antioxidant reactions^14^. This compound plays a critical role in modulating the antioxidant system during plant growth by affecting the transcription of genes responsible for encoding antioxidant enzymes^15^. GABA application also has a significant impact on respiratory metabolism, leading to changes in the activity of numerous enzymes within the tricarboxylic acid (TCA) cycle^16^.

Calcium (Ca) is an essential macronutrient that plays a critical role in maintaining the structural integrity of cell walls, preserving membrane stability, and orchestrating cellular signaling processes in plants^17^. Its presence enhances the strength of cell walls by forming bonds with pectins, thus increasing rigidity and firmness, which provides essential mechanical support to plants^18,19^. In addition, the presence of Ca inhibits the activity of polygalacturonase enzymes, facilitating the maintenance of the middle lamella^20^. Furthermore, this essential macronutrient can modulate the activities of ACC synthase and ACC oxidase, leading to a reduction in endogenous ethylene production by the plant, thus slowing the process of senescence. A study by Islam, et al.^21^ showed an increase in antioxidant responses in tomato plants after the application of exogenous Ca. Due to these beneficial properties, the application of Ca salts, such as calcium chloride, calcium phosphate, calcium citrate, calcium oxide, and calcium lactate, has been reported to significantly prolong the vase life of cut flowers in various species, including *Heliconia* spp^22^, Gerbera^23^, and Gladiolus^24^. Ca has also been shown to delay senescence of cut roses by protecting both membrane phospholipids and membrane proteins from degradation while reducing ethylene production^25^.

There is limited existing literature on the combined use of GABA and Ca, especially regarding their influence on the antioxidant responses of rose flowers. Therefore, the main objective of this study was to evaluate the effects of GABA and Ca treatments, both individually and in combination, on the antioxidant responses of Jumilia rose cut flowers, with the ultimate goal of prolonging their vase life.

## Materials and methods

### Plant materials and experimental setup

The experiment was carried out in a factorial design within a completely randomized setup with three replications and five plants per replication, at the hydroponic greenhouse facilities of Sida Rose Company (Yasuj, Kohgiluyeh and Boyer-Ahmad Province, Iran, 2022).

*Rosa hybrid* cv. Jumilia grafted on Natal Briar rootstock was selected for the study, which were purchased from a local commercial producer that is approved by the Iranian medicinal plants association. These grafted plants were planted in 100 cm × 40 cm plastic pots, filled with a 1:1 ratio cocopeat/perlite (v/v). To ensure optimal growth conditions, the plants were fertigated with a carefully formulated nutritional solution, administered via an open drip irrigation system. The nutrient solution was delivered to the plants five times daily at 2 h intervals. Throughout the growth period, standard horticultural practices were employed, including pruning, pest and disease management, and branch bending. The greenhouse maintained average day/night temperatures of 24 ± 4/15 ± 2 °C and 40–60% relative humidity. Additional information about the water quality and nutrient solution composition can be found in Tables 1 and 2, respectively.

**Table 1:** The characteristics of the tap water used for fertigation and treatment preparation.

Minerals (meq/L)						SSP	SAR	TH	T.A mg/L	EC µS/cm	pH
Ca^+2^	Mg^+2^	Cl^-^	SO_4_^–2^	CO_3_^–2^	HCO_3_^–2^						
3.40	1.10	0.40	1.01	0.00	3.40	2.58	0.08	225	170	401	7.23

meq/L											
Na^+^	K^+^	SO_4_^2-^	B	Fe	NH_4_^+^	PO4_3_^-^	NO_3_^-^	Zn	Cu	Al mg/L	Mn Mg/l
0.12	1.17	0.218	0.08	0.019	0.13	0.56	15.01	0.17	0.01	0.03	0.011

*SSP* soluble sodium percentage, *SAR* sodium adsorption ratio, *TH* total hardness, *EC* electrical conductivity.

**Table 2:** The chemical fertilizer used in the nutrient solution of the plants based on greenhouse conditions.

Fertilizers	Tank A: to prepare 2500 L of nutrient solution (g)	Fertilizers	Tank B: to prepare 2500 L of nutrient solution (g)
Ammonium nitrate	7000	Mono-potassium phosphate	10,500
Potassium nitrate	13,000	Potassium nitrate	13,000
Calcium nitrate	40,000	Magnesium nitrate	11,000
Iron chelate	2800	Ammonium nitrate	7000
pH	5–5.5	Zinc	170
EC	1600–1800	Sodium molybdate	7
		Boric acid	100
		Copper sulfate	75
		Nitric acid	3 lit

### Treatments

The experiment involved two factors: the application of γ-amino butyric acid (GABA) at four different concentrations (0, 20, 40, and 60 mM), calcium chloride (CaCl_2_) at three levels (0, 0.75, and 1.5%): To apply these treatments, a hand-sprayer was used to evenly spray GABA and CaCl_2_ solutions to the plants, ensuring that the flowers were thoroughly wet to the point of runoff. Control flowers were sprayed solely with distilled water. This procedure was repeated three times, each spaced 7 days apart, leading up to the harvest of the flowers. Upon reaching the commercial harvest stage, the flowers were carefully cut and placed in containers filled with water. These containers were stored in a room where the temperature remained at an average of 24 ± 4/15 ± 2 °C during the day and night, while the relative humidity was maintained within the range of 40–60%. In this experiment, 15 rose branches were considered for each treatment, 10 branches for the flower vase life and 5 branches for other tests (biochemical and enzymatic).

### Enzyme extraction

First, 0.5 g of petals were frozen and subsequently ground into a fine powder using liquid nitrogen and a mortar and pestle. The resulting powder was then homogenized in a 2 mL extraction buffer consisting of 50 mM potassium phosphate buffer at pH 8.0. To this buffer, 10% (w/v) polyvinylpyrrolidone (PVP), 0.1 mM ethylenediaminetetraacetic acid (EDTA), and 1 mM dithiothreitol (DTT) were added. The homogenate was centrifuged at 10,000 × g for 30 min, maintaining a temperature of 4 °C. Following centrifugation, the resulting supernatants were carefully collected.

### Superoxide dismutase activity (SOD)

To determine the activity of superoxide dismutase (SOD, EC 1.15.1.1), the following procedure was followed: 0.1 mL of the enzymatic extract was added to a tube containing a mixture of 13 mM L-methionine, 25 mM nitroblue tetrazolium chloride (NBT), 0.1 mM EDTA, 50 mM sodium carbonate, and 2 mM riboflavin, all dissolved in a 50 mM phosphate buffer at pH 7.8, as described by Dhindsa, et al.^26^. The tube was then placed under two 15 W fluorescent lamps for a duration of 15 min. A control sample, without the enzyme, was also prepared to determine the maximal color change. To stop the reaction, the lights were switched off, and the tubes were kept in the dark. Additionally, a non-irradiated complete reaction mixture served as a blank. The absorbance was measured at 560 nm spectrophotometrically, and one unit of enzyme activity was defined as the amount of enzyme required to reduce the absorbance reading to 50% compared to the tubes lacking the enzyme. The SOD activity was expressed as units per milligram of protein per minute.

### Catalase activity

Catalase (CAT, EC 1.11.1.6) activity was assessed spectrophotometrically following the method outlined by Chance and Maehly^27^. This involved monitoring the decrease in absorbance at 240 nm attributed to the consumption of H_2_O_2_. A reaction mixture of 1 mL was prepared, comprising 50 mM potassium phosphate buffer at pH 7.0 and 15 mM H_2_O_2_. The reaction was initiated by introducing 50 μL of the crude extract into this solution. The CAT activity was quantified and expressed as units, μmol of H_2_O_2_ consumed per minute, per milligram of protein.

### Peroxidase activity

The guaiacol peroxidase (POD, EC 1.11. 1.7) activity was determined as follows: A 50 μL portion of the crude enzyme preparation was added to a 2 mL solution containing 50 mM potassium phosphate buffer (pH 7.0), 13 mM guaiacol, and 5 mM H_2_O_2_. The increase in absorbance, resulting from the oxidation of guaiacol (with an extinction coefficient of 26.6 mM^-−1^ cm^−1^), was monitored at 470 nm for one minute. The peroxidase activity was quantified and expressed in units, μmol guaiacol oxidized per minute, per milligrams of protein^28^.

### Polyphenol oxidase and ACC synthase activity

Polyphenol oxidase (PPO, EC1.10.3.1) activity was determined following the protocol established by Kumar and Khan^29^. The assay mixture for PPO consisted of 2 mL of 0.1 M phosphate buffer at pH 6.0, 1 mL of 0.1 M catechol, and 0.5 mL of the enzyme extract. This mixture was incubated at 25 °C for 5 min, and the reaction was halted by adding 1 mL of 2.5 N H2SO4. The absorbance of the resulting purpurogallin was measured at 495 nm. PPO activity is expressed in units (μmol catechol oxidized per minute) per mg protein.

The ACC synthase activity in petals was measured according to the method of Jiang et al.^30^ with some modification. After preparation the samples, the ACC was converted to ethylene. The ethylene in the headspace was then measured using a Shimadzu GC 9A gas chromatograph. A unit of ACC synthase activity was defined as the amount of enzyme that catalyzed the formation of 1 nmol of ACC per hour under the stated assay conditions.

### Malondialdehyde content

Malondialdehyde (MDA) content was assessed following the thiobarbituric acid (TBA) reaction, as originally outlined by Ali, et al.^31^, with minor adjustments. Initially, 200 mg of petal samples were homogenized in 2 mL of 0.1% trichloroacetic acid and subjected to centrifugation at 10,000 × g for 15 min. Next, 1 mL of the resulting supernatant was combined with 2.5 mL of 0.5% thiobarbituric acid in 20% trichloroacetic acid and subjected to incubation in hot water at 95 °C for 30 min. The reaction was promptly terminated by cooling the mixture on ice, followed by centrifugation at 10,000 × g for 30 min. The absorbance was measured at 532 and 600 nm. The concentration of MDA was determined by subtracting the nonspecific absorption at 600 nm from the absorption at 532 nm, using an absorbance coefficient of extinction (155 mM^−1^ cm^−1^).

### Vase life

Sharp secateurs were used to cut flowers. The selection criteria included choosing flowers at the tight bud stage, characterized by fully developed coloration while the petals remained tightly closed, yet to begin unfolding. For the assessment of vase life, a total of 15 flower stems were harvested from each treatment group. These stems were then recut to a standardized length of 50 cm and placed individually in containers filled with water. The containers were maintained at room temperature, consistently set at 25 °C, with relative humidity levels ranging between 85 and 90%. In accordance with the criteria established by Jowkar, et al.^32^: the vase life was carefully monitored and evaluated on a daily basis. The end of the vase life was determined when either the neck of the flower was bent or the five outermost petals showed visible signs of wilting.

### Total flavonoid content

To determine the total flavonoid content in the methanolic extract solution, the aluminum chloride assay, as described by Tohidi et al.^34^, was employed. An aliquot of 125 μl from the extract solution was mixed with 75 μl of 5% NaNO_2_ solution. The mixture was then allowed to stand for 5 min before the addition of 150 μl of aluminum chloride (10%) solution. Following this, 750 μl of NaOH solution (1 M) was incorporated, and the final volume of the mixture was adjusted to 2500 μl using deionized water. After incubating the mixture for 15 min, the absorbance was measured at 510 nm using a spectrophotometer. The total flavonoid content was quantified and expressed as milligrams of quercetin equivalent per gram of fresh weight.

#### Total phenolic content

Initially, 1.25 g of petal samples were combined with 25 mL of 80% methanol in an orbital shaker set at 150 rpm and maintained at a temperature of 25 °C. The mixture was allowed to shake for a duration of 24 h. The total phenolic compounds in the resulting methanolic extract solution was determined using the Folin Ciocalteu method, following the protocol outlined by Tohidi, et al.^34^, with minor adjustments. Specifically, 0.5 mL of the filtered methanolic extract was added to a test tube containing a mixture of 2.5 mL of the Folin Ciocalteu reagent (diluted tenfold) and 2 mL of 7.5% sodium carbonate. The contents of the test tube were thoroughly mixed. After heating this mixture at 45 °C for 15 min, the absorbance was measured at 765 nm using a spectrophotometer. Gallic acid was utilized as the standard for quantifying the total phenolic content. The resulting data were expressed as milligrams of gallic acid equivalent per gram of fresh weight.

### Total antioxidant activity

The total antioxidant activity was determined using the DPPH (2,2-diphenyl-picryl-hydrazyl) radical degradation method. Specifically, 10 μL of petal extract was combined with 4 mL of distilled water in test tubes, followed by the addition of 1 mL of a 250 μM DPPH solution. Subsequently, the test tubes were left undisturbed in darkness for a 30-min incubation period. Afterward, the absorbance was measured at 517 nm using a spectrophotometer. The antioxidant activity was then calculated as the percentage of inhibition relative to the control, employing the following formula^35^: .

$$ {\text{Antioxidant}}\% = \left( {{\text{A}}\;{\text{blank}} - {{{\text{A}}\;{\text{sample}}} \mathord{\left/ {\vphantom {{{\text{A}}\;{\text{sample}}} {{\text{A}}\;{\text{blank}}}}} \right. \kern-0pt} {{\text{A}}\;{\text{blank}}}}} \right) \times 100 $$

where A blank is the absorbance of the control reaction, and A sample is the absorbance of the test compound in the sample.

### Petal carotenoid content

Petals (0.5 g) were extracted in 5 mL of acetone (80%), then centrifuged (8000 × *g*) for 10 min. The supernatant was used to make a final volume of 100 mL of the petal extract. Extraction of petal tissue with the buffer continued three times. The absorbance of the extract was read at 470 nm with a spectrophotometer and 80% acetone was used as a blank. Carotenoid content of petal tissue was determined using the following formula^36^: .

$$ \begin{aligned} {\text{Carotenoid}}\;{\text{content}}\;{\text{of}}\;{\text{petaltissue}}\;\left( {{\text{mg}}{\text{.g}}^{{ - 1}} {\text{FW}}} \right){ = } & \left( {{\text{Absorbance}}\;{\text{at}}\;{470}\;{\text{nm}}} \right){\text{/Specific}}\;{\text{absorption}}\;{\text{coefficient}}\;{\text{of}}\;{\text{carotenoids)}} \\ {*}\left( {{\text{Volume}}\;{\text{of}}\;{\text{extract}}} \right){/}\left( {{\text{Weight}}\;{\text{of}}\;{\text{petaltissue}}} \right) \\ \end{aligned} $$

It should be noted that all the protocols used in this study are in accordance with national, and international guidelines and legislation.

### Statistical analysis

Data were analyzed by SAS (9.4M7), and means were compared using Duncan’s multiple range tests at the 5% probability level. When the interaction between treatments was significant, as determined by ANOVA, main effects were not presented. (The raw data is attached as a supplementary file).

## Results

### Vase life

**Figure 1:**
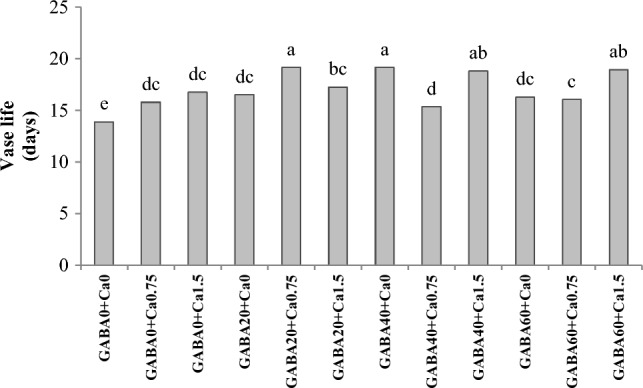
Interaction effect of GABA and CaCl2 on vase life of Jumilia rose. Columns with different letters represent significant differences at 5% probability using Duncan’s multiple range test. Vertical bars indicate standard errors (n=3).

In all samples, an increase in vase life was observed, and this increase was significantly higher than that of the control group (Fig. 1). The longest vase life duration was noted in plants that were treated with combinations of GABA (20 mM) and CaCl_2_ (0.75%), GABA (60 mM) and CaCl_2_ (1.5%), or GABA (40 mM) applied individually.

### Total protein content

**Figure 2:**
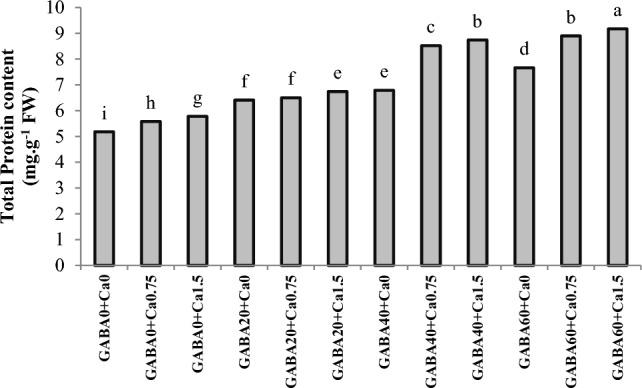
Interaction effect of GABA and CaCl2 on total protein content in petals of Jumilia rose. Columns with different letters represent significant differences at 5% probability using Duncan’s multiple range test. Vertical bars indicate standard errors (n=3).

In Fig. 2, the interaction effects of different concentrations of GABA (0, 20, 40, 60 mM) and CaCl_2_ (0, 0.75%, 1.5%) on the total protein content in rose petals are depicted. An increase in this parameter was observed across all treatments, with all treated samples exhibiting significantly higher protein content compared to the control group. The application of CaCl_2_ alone increased this parameter; however, the effect of GABA was more pronounced. In samples that received GABA treatments (alone), the protein content was higher than in those treated with CaCl_2_ alone. A synergistic effect was observed between these two compounds, as the combined effect was greater than the sum of their separate effects. Notably, in plants treated with the highest levels of GABA (60 mM) and CaCl_2_ (1.5%), the protein content was elevated to 9.17 mg. g^−1^ FW, representing an approximately 1.77-fold increase when compared to the control samples (5.18 mg. g^−1^ FW).

### Flavonoid content, carotenoids and antioxidant activity

Table 3 demonstrates the interaction effect of different levels of GABA and CaCl_2_ on non-enzymatic antioxidants and total antioxidant activity in rose petals. An increase in flavonoid content in petals was observed with the elevation of both GABA and CaCl_2_ levels. The highest concentration of flavonoids (21.17 mg. g^−1^ FW) was obtained when roses were treated with a CaCl_2_ solution at a 1.5% concentration and GABA at 40 mM. A significant increase in petal carotenoid concentration was also noted in response to CaCl_2_ and/or GABA, whether applied individually or in combination. In all treated plants, this parameter was significantly higher compared to the control group, with samples treated with CaCl_2_ (1.5%) and GABA (60 mM) recording 29.53 mg. g^−1^ FW carotenoids, representing an increase of over 2.5 times in comparison to the control group. A dose-dependent increase in total phenolic compounds in the petals was observed with the application of GABA and CaCl_2_, reaching 26.55 mg. g^−1^ FW at the highest treatment levels. This concentration was 1.62 times higher than that of the control group, which displayed 16.36 mg. g^−1^ FW. Additionally, a significant enhancement in the antioxidant activity of the petals was observed with the application of CaCl_2_ and/or GABA, whether applied individually or in combination. The control group exhibited significantly lower antioxidant activity (26.61%) compared to all other treatments. Generally, the highest levels of carotenoids, phenolic content, and antioxidant activity were observed when using the highest combined concentrations of GABA and CaCl_2_.

**Table 3:** Interaction effect of GABA and Cacl_2_ on the concentration of non-enzymatic antioxidants and the antioxidant activity in Jumilia rose’s petals. Means (n = 3) followed by the same letters within columns are not different at 5% probability using Duncan’s test.

Treatments		Flavonoids	Carotenoids	Phenolic compounds	Antioxidant activity
CaCl_2_ (% w/v)	GABA (mM)	(mg g^-1^ FW)			(%)
**0**	**0**	11.21 k	10.81 l	16.36 k	26.61 k
**0.75**		11.25 k	11.66 k	16.48 k	27.18 j
**1.5**		11.51 j	12.72 j	17.56 j	27.75 i
**0**	**20**	13.41 i	16.03 i	19.91 i	29.69 h
**0.75**		14.12 h	16.71 h	21.69 g	30.65 g
**1.5**		14.66 g	17.26 g	22.11 f.	31.3 f.
**0**	**40**	18.62 e	18.35 f.	21.32 h	31.15 f.
**0.75**		20.24 b	25.16 d	24.07 d	37.85 d
**1.5**		21.17 a	26.33 c	24.61 c	39.1 c
**0**	**60**	17.91 f.	20.26 e	23.12 e	33.51 e
**0.75**		19.26 d	28.18 b	25.71 b	41.26 b
**1.5**		19.77 c	29.53 a	26.55 a	42.51a
Significance					
GABA		**	**	**	**
CaCl_2_		**	**	**	**
CaCl_2_ × GABA		**	**	**	**

Significants values are in bold.

### Enzymatic antioxidant and polyphenol oxidase activities

Table 4 shows the interaction effects of different levels of GABA and CaCl_2_ on enzymatic antioxidant and polyphenol oxidase activities in rose petals. SOD activity exhibited a significant rise in response to higher concentrations of both GABA and CaCl_2_. The SOD activity in roses treated with a combination of CaCl_2_ (1.5%) and GABA (60 mM) reached 7.86 Units. mg^−1^ protein min^−1^, marking an approximately 2.93-fold increase compared to the control group (2.68 Units. mg^−1^ protein min^−1^). Similar trend was observed for the CAT activity. When roses were treated with CaCl_2_ (1.5%) and GABA (60 mM), the CAT activity reached 12.1 Units. mg^−1^ protein min^−1^, nearly tripling the activity level observed in the control group. The POD activity also followed a similar pattern of increase with the rise in CaCl_2_ and GABA concentrations. Samples treated with the highest levels of both compounds displayed a POD activity of 4.88 Units. mg^−1^ protein min^−1^ which was higher than all other treatments. In contrast, PPO activity showed a reverse response to elevated CaCl_2_ and GABA levels. The combination of CaCl_2_ (1.5%) and GABA (60 mM) resulted in a PPO activity of 1.84 Units. mg^−1^ protein min^−1^, which was lower than all other treatments.

**Table 4:** Interaction effect of GABA and CaCl_2_ on the enzymatic antioxidants and ACC synthase activity in Jumilia rose’s petals. Means (n = 3) followed by the same letters within columns are not different at 5% probability using Duncan’s test.

Treatments		SOD	CAT	POD	PPO	ACC synthase activity
CaCl_2_ (% w/v)	GABA (mM)	U mg^-1^ protein min^-1^				mmol g^−1^ FW
0	**0**	2.68 ij	4.13 j	2.17 h	4.84 ab	8.18a
0.75		2.55 j	4.66 i	2.22 h	4.96 a	7.77b
1.5		2.76 i	4.79 i	2.46 g	4.75 b	7.68b
0	**20**	3.68 h	5.92 h	2.97 f.	4.3 c	6.2d
0.75		4.27 g	6.69 g	3.08 f.	3.18 f.	5.5e
1.5		4.59 f.	7.03 f.	3.35 e	2.92 g	7.49c
0	**40**	4.37 g	7.07 f.	3.33 e	4.02 d	4.86g
0.75		6.66 d	10.44 d	4.17 c	2.21 h	3.51j
1.5		7.17 c	10.96 c	4.32 c	2.07 hi	3.24k
0	**60**	5.05 e	7.98 e	3.84 d	3.71 e	5.1f.
0.75		7.48 b	11.59 b	4.55 b	1.96 ji	4.52h
1.5		7.86 a	12.1 a	4.88 a	1.84 j	3.96i
Significance						
GABA		**	**	**	**	**
CaCl_2_		**	**	**	**	**
CaCl_2_ × GABA		**	**	**	**	**

Significants values are in bold.

### ACC synthase activity

In the present study the application of GABA and CaCl_2_ significantly (P < 0.01) increased the ACC synthase activity in the petals of the cut rose flowers (Table 4). The results showed that the foliar pre-harvest application of GABA and CaCl_2_ reduced the ACC synthase activity. The highest and lowest of ACC synthase activity was obtained in the rose plants treated with GABA at 40 mM and CaCl_2_ at 1.5 and 0.75% rates and the untreated flowers (3.57, 3.96, and 8.18 nmol g-1 FW, respectively). In fact, the combined application of GABA and CaCl_2_ decreased ACC synthase activity by 151% compared to the untreated plants (Table 4).

### MDA

**Figure 3:**
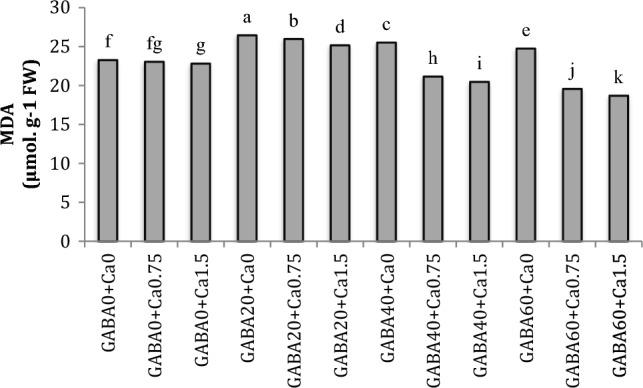
Interaction effect of GABA and CaCl2 on MDA content in petals of Jumilia rose. Columns with different letters represent significant differences at 5% probability using Duncan’s multiple range test. Vertical bars indicate standard errors (n=3).

The petal MDA content exhibited a significant decrease in roses subjected to higher levels of GABA and CaCl_2_. Specifically, in samples treated with CaCl_2_ (1.5%) and GABA (60 mM), the MDA content in the petals decreased by 20% relative to the control group (Fig. 3).

### Correlation between parameters

**Figure 4:**
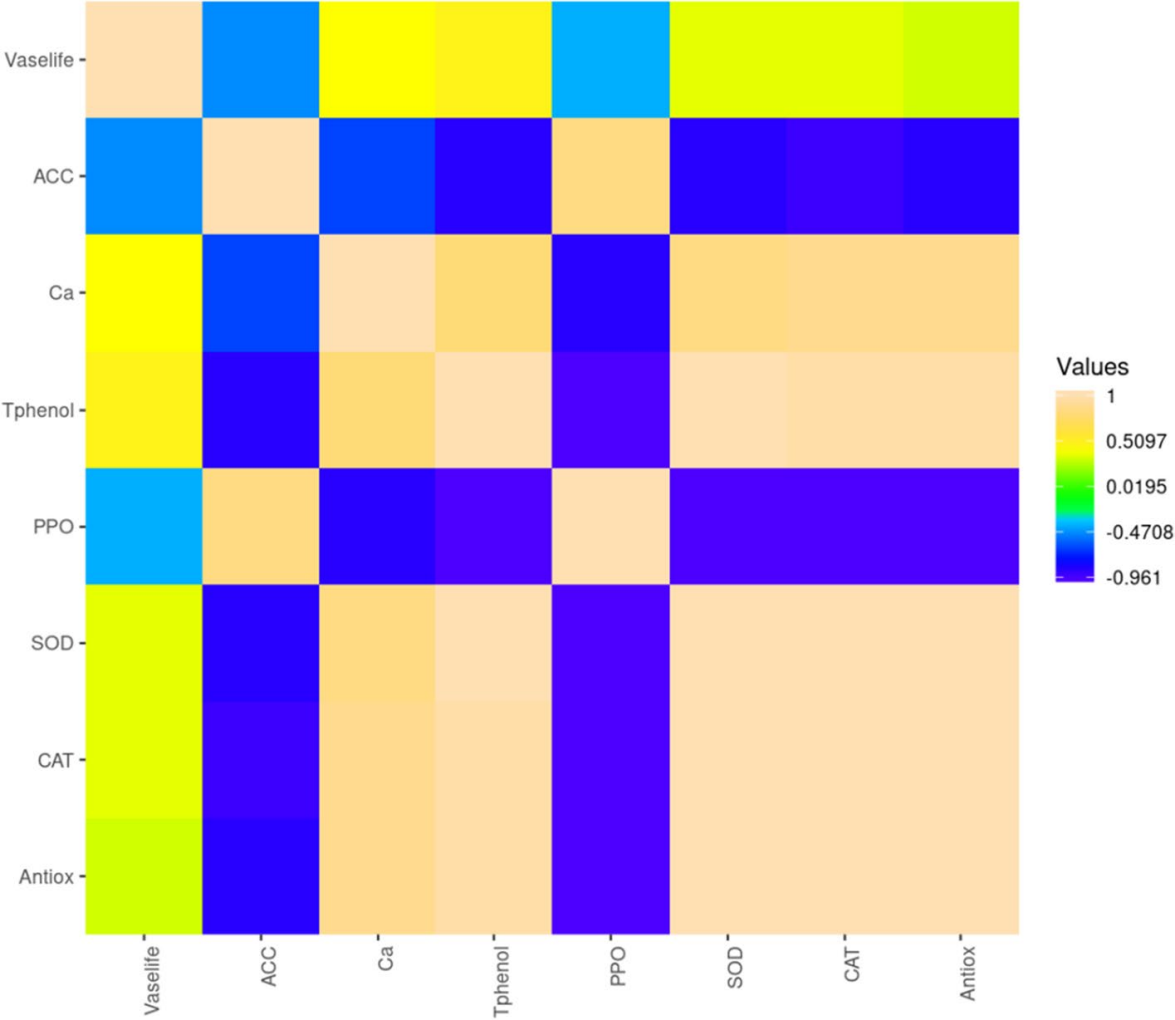
Heat-map matrix of the correlation coefficients between the vase life and major traits in petals rose flower treated with different concentrations of GABA and CaCl2. Each square indicates r (Pearson’s correlation coefficient of a pair of traits).

Vase life showed no significant correlation with the parameters studied. It displayed a non-significant negative correlation with ACC and PPO, as well as a non-significant positive relationship with other characteristics. ACC had a highly significant positive correlation with PPO and a significant negative correlation with Ca, total phenolic compounds, SOD, CAT, and total antioxidant activity. Total phenolic compounds exhibited a negative correlation with ACC and PPO, but showed a significant positive correlation with ACC, SOD, CAT, and total antioxidant activity. PPO had a highly significant positive correlation with ACC but significant negative correlations with Ca, total phenolic compounds, SOD, CAT, and total antioxidant activity. SOD showed a highly significant positive correlation with Ca, Total Phenol, CAT, and total antioxidant activity, while maintaining a significant negative correlation with ACC and PPO. CAT had a highly significant negative correlation with ACC and PPO, and a significant positive correlation with other parameters, excluding vase life. Total antioxidant activity also demonstrated a highly significant negative correlation with ACC and PPO, along with a significant positive correlation with Ca, total phenolic compounds, SOD, and CAT (Table 5 and Fig. 4).

**Table 5:** Pearson’s correlation coefficients in Jumilia rose’s petals treated with different concentrations of GABA and Cacl_2._ *. Correlation is significant at the 0.05 level (2-tailed). **. Correlation is significant at the 0.01 level (2-tailed).

	Vase life	ACC synthase activity	Ca	Total phenolic compounds	PPO	SOD	CAT	Total antioxidant activity
Vase life	1							
ACC synthase activity	− 0.464	1						
Ca	0.393	− 0.619*	1					
Total phenolic compounds	0.460	− 0.871**	0.766**	1				
PPO	− 0.389	0.810**	− 0.858**	− 0.949**	1			
SOD	0.348	− 0.880**	0.838**	0.965**	-0.961**	1		
CAT	0.350	− 0.887**	0.846**	0.960**	-0.951**	0.998**	1	
Total antioxidant activity	0.318	− 0.859**	0.857**	0.946**	− 0.937**	0.993**	0.996**	1

## Discussion

Our results demonstrated that exogenous application of both GABA and CaCl_2_ can synergistically enhance the enzymatic/non-enzymatic antioxidant response in rose flowers. The increased flavonoid, carotenoid, phenolic, and antioxidant activity levels as well as higher SOD, CAT, and POD activity observed with combined GABA and CaCl_2_ application align with previous investigations showing similar antioxidant-elevating properties of these compounds. A number of investigations have ascertained that GABA can induce the activation of the antioxidant defense system in response to abiotic stresses, thereby mitigating oxidative damage caused by the production of reactive oxygen species (ROS)^37–39^. Subsequent to the application of exogenous GABA, a significant rise in the activity of enzymatic antioxidants within leaves is observed. This is accompanied by a reduction in the accumulation of ROS, consequently enhancing the resilience of seedlings to adverse environmental conditions^40–42^. In an in vitro experiment, it was demonstrated that GABA and proline reduced ROS levels. GABA exhibits a superior capacity for the removal of superoxide anions (O^2-^) and hydrogen peroxide (H_2_O_2_) compared to proline^43^.

Ca, through its binding affinity with the phospholipid bilayer, exerts regulatory control over membrane architecture, signaling cascades, and membrane functionality. Consequently, this involvement facilitates the reinforcement and improvement of structural integrity within membrane organelles in plants mitigating adverse environmental changes^44^. Previous studies have documented the beneficial influence of exogenous Ca application in alleviating environmental changes in plants. These advantageous effects have been attributed to physiological mechanisms, which include osmotic adjustment, improved antioxidant (enzymatic/non-enzymatic) responses, modulation of Na and K ion homeostasis, enhancement of proline accumulation, and facilitation of root and shoot growth^45–47^. In addition, Ca assumes a pivotal role as a secondary messenger in orchestrating plant responses to adverse environmental conditions^48^. There exists an interrelationship between this essential macronutrient and GABA concerning the activation of plant antioxidant responses^49^. In essence, the accumulation of GABA under both biotic and abiotic stress conditions is intricately linked to the regulation of intracellular Ca^2+^ and its interaction with calmodulin (CaM).

Under non-optimal conditions such as drought, salinity, and temperature stress, plants exhibit an elevation in intracellular Ca^2+^ levels. This surge in Ca^2+^ concentration serves as a stimulatory cue for Ca^2+^/CaM to activate glutamate decarboxylase (GAD), culminating in the accumulation of GABA. Trobacher, et al.^50^ reported that the regulatory network involving Ca^2+^/CaM, GAD, and GABA depends on MdGAD1 and MdGAD2 in apple. Their activity and spectral properties are modulated by Ca^2+^/CaM balance and acidic pH. Moreover, the application of exogenous Ca has been demonstrated to activate GAD, thereby promoting the accumulation of GABA in carrots and pears^51–53^. This collective body of research underscores the interplay between Ca signaling and the regulation of GABA levels in plants, particularly in response to challenging environmental conditions.

Our findings indicated that PPO activity decreased in response to elevated CaCl_2_ and GABA levels. The reduced PPO activity provides evidence that GABA and CaCl_2_ mitigate oxidative damage by enhancing antioxidants as higher PPO is associated with senescence, decay, and quality loss in flowers^54–56^.

Our findings indicate applying GABA and Ca could help maintain postharvest quality as treated samples exhibited prolonged vase life, diminished petal MDA content, and higher petal protein concentration. This was in agreement with previous investigations. Gerbera cut flowers, when subjected to a 1 mM GABA treatment, displayed a reduction in electrolyte leakage, H_2_O_2_, MDA, lipoxygenase (LOX), and phospholipase D (PLD) activity. Simultaneously, these treated flowers exhibited an increase in proline content and enhanced antioxidant enzyme activities^6,7^. The application of GABA, both pre- and post-harvest, in the vase solution at ambient temperatures, resulted in enhanced quality and extended vase life of rose cut flowers, as reported by Mirzaei Mashhoud, et al. ^5^. Similar finding were reported by Babarabie, et al.^13^. They observed an improved vase life of tuberose flowers treated with GABA. In a study on *Narcissus tazetta* cv. ‘Shahla-e-Shiraz’, it was observed that the activity of PPO experienced significant inhibition in the presence of GABA. Moreover, GABA played a crucial role in enhancing the relative water content of narcissus petals during storage by mitigating alterations in the cellular membrane stability index^12^. Abdolmaleki, et al.^57^ proposed the use of CaCl_2_ as a method for enhancing the postharvest longevity of roses. Their research revealed that ‘Dolce Vita’ roses treated with a CaCl_2_ and/or salicylic acid solutions exhibited significantly prolonged vase life. Our findings and this observed improvement can be attributed to the previously mentioned interplay between Ca and GABA, orchestrating plant responses under stressful conditions. The synergistic action of Ca and GABA appears to optimize these responses. In this context, the bolstered antioxidant response induced by Ca and GABA can effectively mitigate the rate of senescence. This results in the preservation of crucial cellular structures such as proteins and membrane functionality for an extended duration, ultimately contributing to the overall longevity and quality of postharvest cut flowers.

In the present study, several correlations were identified among the studied parameters. SOD and CAT were positively correlated. SOD plays a crucial role in the first line of defense against oxidative stress by converting superoxide anions into H_2_O_2_. Hydrogen peroxide is then efficiently converted into water and molecular oxygen by CAT, acting as a potent ROS scavenger^58^. SOD exhibited a strong positive correlation with total antioxidant activity, underscoring its significant role in the plant's antioxidant system^59^. Conversely, PPO showed a negative correlation with total antioxidant activity, CAT, and SOD, aligning with previous studies that reported a decrease in PPO activity following abiotic stress, associated with improved antioxidant capacity^35^. Total phenolic compounds were negatively correlated with PPO, potentially due to the enzymatic activity of PPO oxidizing phenolic compounds^60^. A positive correlation was observed between total phenolic compounds and enzymatic antioxidants—SOD, CAT, and total antioxidant activity. This suggests that polyphenols play a role in the plant's mechanism against ROS, similar to SOD and CAT, indicating that higher polyphenolic compounds may lead to increased total antioxidant activity^58,61^. Ca exhibited positive correlations with SOD, CAT, and total antioxidant activity, while showing a negative correlation with PPO. Calcium is known to play a crucial role in the activation of antioxidant enzymes such as SOD and CAT^62^. Ca has an inhibitory effect on PPO activity^63^. This can result in an increased content of polyphenolic compounds and improved antioxidant responses, thereby enhancing vase life.

## Conclusion

In conclusion, our findings demonstrated that the combined application of GABA and CaCl_2_ can synergistically enhance antioxidant activity and postharvest quality in cut rose flowers. Our results also revealed diminished PPO activity and MDA content along with higher protein levels and an extended vase life duration, indicating GABA and CaCl_2_ help mitigate the senescence. Overall, this research highlights the potential of using GABA and CaCl_2_ treatment as an effective postharvest strategy to prolong quality and vase life of cut roses by enhancing antioxidant capacity and delaying senescence.

### Supplementary Information

Supplementary Tables.

## Data Availability

The datasets used and/or analyzed during the current study available from the corresponding author on reasonable request.
