# Supplementary material for: Improving postharvest quality and vase life of cut rose flowers by pre-harvest foliar co-applications of γ-aminobutyric acid and calcium chloride
Source: Sci Rep. 2024 Jun 24;14:14520. doi: 10.1038/s41598-024-64021-8 (PMC11196717; doi:10.1038/s41598-024-64021-8)
Supplement: Supplementary file 1 — Supplementary Tables. [file 41598_2024_64021_MOESM1_ESM.docx]

| Treatments | | Vaselife (day) | ACC synthase activity (mmol g-1 FW) | Flavonoids | Phenolic compounds | Carotenoids (mgg-1 FW) | Total protein content | MDA (µmol.g-1FW) | SOD (U mg-1 protein min-1) |
| --- | --- | --- | --- | --- | --- | --- | --- | --- | --- |
| CaCl2  (% w/v) | GABA (Mm) |  |  |  |  |  |  |  |  |
| 0 | 0 | 13.85^e^±1.53 | 8.18^a^±1.68 | 11.21^k^±1.12 | 16.36^k^±2.42 | 10.81^l^±2.15 | 5.18^i^±0.37 | 23.26^f^±1.07 | 2.68^ij^±0.42 |
| 0.75 |  | 15.75^cd^±2.15 | 7.77^b^±0.95 | 11.25^k^±0.97 | 16.48^k^±1.87 | 11.66^k^±0.87 | 5.58h±0.63 | 23.04^fg^±2.35 | 2.55^j^±0.64 |
| 1.5 |  | 16.7^cd^±1.12 | 7.68^b^±0.87 | 11.51^j^±1.18 | 17.56^j^±2.18 | 12.72^j^±2.57 | 5.78^g^±0.54 | 22.8^g^±1.28 | 2.76^ij^±0.23 |
| 0 | 20 | 16.45^cd^±3.24 | 6.2^d^±0.55 | 13.41^i^±1.64 | 19.91^i^±2.24 | 16.03^i^±1.25 | 6.41^f^±1.79 | 26.44^a^±3.24 | 3.68^h^±0.54 |
| 0.75 |  | 19.1^a^±1.08 | 5.5^e^±0.74 | 14.12^h^±2.24 | 21.69^g^±3.12 | 16.71^h^±3.04 | 6.5^f^±0.34 | 25.96^b^±2.6 | 4.27^g^±1.14 |
| 1.5 |  | 17.21^bc^±1.37 | 7.49^c^±0.61 | 14.66^g^±1.67 | 22.11^f^±2.54 | 17.26^g^±2.15 | 6.74^e^±0.88 | 25.15^d^±2.07 | 4.59^f^±0.64 |
| 0 | 40 | 19.1^a^±0.65 | 4.86^hg^±0.72 | 18.62^e^±2.08 | 21.32^h^±3.68 | 18.35^f^±1.47 | 6.79^e^±1.49 | 25.51^c^±1.87 | 4.37^g^±0.61 |
| 0.75 |  | 15.3^d^±1.15 | 3.51^i^±0.54 | 20.24^b^±2.47 | 24.07^d^±3.04 | 25.16^d^±2.24 | 8.52^c^±0.91 | 21.14^h^±0.84 | 6.66^d^±0.38 |
| 1.5 |  | 18.74^ab^±2.11 | 3.24^k^±0.94 | 21.17^a^±1.07 | 24.61^c^±2.64 | 26.33^c^±2.66 | 8.74^b^±0.24 | 20.47^i^±1.65 | 7.17^c^±0.39 |
| 0 | 60 | 16.23^cd^±3.17 | 5.1^f^±0.83 | 17.91^f^±1.34 | 23.12^e^±4.13 | 20.26^e^±1.95 | 7.66^d^±0.59 | 24.74^e^±2.12 | 5.05^e^±0.77 |
| 0.75 |  | 16^c^±0.98 | 4.52^h^±1.04 | 19.26^d^±1.38 | 25.71^b^±3.17 | 28.18^b^±1.69 | 8.9^b^±1.37 | 19.56^j^±3.21 | 7.48^b^±0.82 |
| 1.5 |  | 18.87^ab^±2.14 | 3.96^i^±0.68 | 19.77^c^±1.45 | 26.55^a^±4.21 | 29.53^a^±1.74 | 9.17^a^±1.65 | 18.69^k^±0.78 | 7.86^a^±0.46 |

**Table S1.** Interaction effect of GABA and Cacl_2_ on the Vaselife, ACC synthase activity, Flavonoids, Phenolic compounds, Carotenoids, Total protein content, MDA, SOD, CAT, POD, PPO and Antioxidant activity in Jumilia rose’s petals. Means (n=3) followed by the same letters within columns are not different at 5% probability using Duncan’s test.

Table S1. Cont.

| Treatments | | CAT  (U mg-1 protein min-1) | POD  (U mg-1 protein min-1) | PPO  (U mg-1 protein min-1) | Antioxidant activity |
| --- | --- | --- | --- | --- | --- |
| CaCl2  (% w/v) | GABA (Mm) |  |  |  |  |
| 0 | 0 | 4.13^j^±0.88 | 2.17^h^±0.34 | 4.84^ab^±0.87 | 26.61^k^±3.26 |
| 0.75 |  | 4.66^i^±1.06 | 2.22^h^±0.23 | 4.96^a^±1.36 | 27.18^j^±2.84 |
| 1.5 |  | 4.79^i^±0.69 | 2.46^g^±0.64 | 4.75^ab^±1.09 | 27.75^i^±4.59 |
| 0 | 20 | 5.92^h^±0.72 | 2.97^f^±0.58 | 4.3^c^±0.75 | 29.69^h^±2.36 |
| 0.75 |  | 6.69^g^±0.43 | 3.08^f^±0.85 | 3.18^f^±0.52 | 30.65^g^±3.45 |
| 1.5 |  | 7.03^f^±0.76 | 3.35^e^±0.76 | 2.92^g^±0.81 | 31.3^f^±0.98 |
| 0 | 40 | 7.07^f^±0.64 | 3.33^e^±0.39 | 4.02^d^±1.48 | 31.15^f^±3.64 |
| 0.75 |  | 10.44^d^±1.54 | 4.17^cd^±0.25 | 2.21^h^±0.94 | 37.85^d^±4.33 |
| 1.5 |  | 10.96^c^±2.01 | 4.32^cd^±0.81 | 2.07^hi^±0.83 | 39.10^c^±3.48 |
| 0 | 60 | 7.98^e^±0.57 | 3.84^d^±0.73 | 3.71^e^±0.67 | 33.51^e^±1.08 |
| 0.75 |  | 11.59^b^±1.69 | 4.55^bc^±0.58 | 1.96^ij^±0.48 | 41.26^b^±3.07 |
| 1.5 |  | 12.10^a^±1.28 | 4.88^a^±0.76 | 1.84^j^±0.22 | 42.51^a^±4.61 |

Table 2: Raw data the effect of GABA and CaCl2 on Improving postharvest quality and vase life of cut rose flowers

| **GABA** | **CaCl2** | **GABA×**  **CaCl2** | **Replication** | **Vase life** | **ACC synthase activity** | **Ca** | **Flavonoids** | **Phenolic compounds** | **Carotenoids** | **Protein** | **MDA** | **SOD** | **CAT** | **POD** | **PPO** | **Antioxidant activity** |
| --- | --- | --- | --- | --- | --- | --- | --- | --- | --- | --- | --- | --- | --- | --- | --- | --- |
| **1** | **1** | **11** | **1** | 13.50 | 8.25 | 5.50 | 11.70 | 16.65 | 11.10 | 5.35 | 23.30 | 2.98 | 4.65 | 2.25 | 4.20 | 27.20 |
| **1** | **2** | **12** | **1** | 17.00 | 8.00 | 6.50 | 12.00 | 16.10 | 10.50 | 5.20 | 22.80 | 2.75 | 4.90 | 2.18 | 4.80 | 26.90 |
| **1** | **3** | **13** | **1** | 16.00 | 7.50 | 8.00 | 12.20 | 18.10 | 13.00 | 6.20 | 22.60 | 2.62 | 5.25 | 2.38 | 5.15 | 27.35 |
| **2** | **1** | **21** | **1** | 17.00 | 5.90 | 6.10 | 13.95 | 19.25 | 15.75 | 6.60 | 26.90 | 3.70 | 5.60 | 2.85 | 3.95 | 30.40 |
| **2** | **2** | **22** | **1** | 19.50 | 5.00 | 7.00 | 14.75 | 22.20 | 16.25 | 6.10 | 26.10 | 4.31 | 7.10 | 3.24 | 3.65 | 31.40 |
| **2** | **3** | **23** | **1** | 18.00 | 8.00 | 8.20 | 15.00 | 21.90 | 17.90 | 7.25 | 25.00 | 4.52 | 7.35 | 3.43 | 3.10 | 32.50 |
| **3** | **1** | **31** | **1** | 20.00 | 5.20 | 7.00 | 19.20 | 21.10 | 17.75 | 6.35 | 25.65 | 4.33 | 6.95 | 3.16 | 3.95 | 30.95 |
| **3** | **2** | **32** | **1** | 16.00 | 2.90 | 9.10 | 20.40 | 24.70 | 26.10 | 9.35 | 21.32 | 6.48 | 11.35 | 4.30 | 1.95 | 38.50 |
| **3** | **3** | **33** | **1** | 19.50 | 4.00 | 9.85 | 22.00 | 25.10 | 27.15 | 9.00 | 20.51 | 7.12 | 11.65 | 4.28 | 1.85 | 39.90 |
| **4** | **1** | **41** | **1** | 17.50 | 4.80 | 6.35 | 18.30 | 22.90 | 20.70 | 8.25 | 24.55 | 5.00 | 9.10 | 3.87 | 3.95 | 34.20 |
| **4** | **2** | **42** | **1** | 17.00 | 5.00 | 9.20 | 18.50 | 25.30 | 29.50 | 9.35 | 19.60 | 7.65 | 12.30 | 4.61 | 1.75 | 41.50 |
| **4** | **3** | **43** | **1** | 18.00 | 4.00 | 11.20 | 18.60 | 27.60 | 30.35 | 9.35 | 18.75 | 8.03 | 12.35 | 4.74 | 2.15 | 43.25 |
| **1** | **1** | **11** | **2** | 16.00 | 9.00 | 6.20 | 12.10 | 15.10 | 12.10 | 4.15 | 23.10 | 2.55 | 3.75 | 2.14 | 5.10 | 26.50 |
| **1** | **2** | **12** | **2** | 14.00 | 8.90 | 7.50 | 10.35 | 17.50 | 12.35 | 4.80 | 22.90 | 2.41 | 5.10 | 2.38 | 6.10 | 28.30 |
| **1** | **3** | **13** | **2** | 15.50 | 8.50 | 8.50 | 11.60 | 16.40 | 11.35 | 6.10 | 23.10 | 2.96 | 4.55 | 2.65 | 4.65 | 29.00 |
| **2** | **1** | **21** | **2** | 15.00 | 5.00 | 4.50 | 12.20 | 19.65 | 15.45 | 7.60 | 26.30 | 3.75 | 6.25 | 3.20 | 4.90 | 28.65 |
| **2** | **2** | **22** | **2** | 18.00 | 7.00 | 8.75 | 12.65 | 20.50 | 17.00 | 7.50 | 25.80 | 4.35 | 6.15 | 3.15 | 3.00 | 31.10 |
| **2** | **3** | **23** | **2** | 16.50 | 8.50 | 9.00 | 14.75 | 21.65 | 18.10 | 6.30 | 24.90 | 4.50 | 6.85 | 3.38 | 2.75 | 28.55 |
| **3** | **1** | **31** | **2** | 20.50 | 6.00 | 7.20 | 17.20 | 20.36 | 19.20 | 6.80 | 25.30 | 4.50 | 7.15 | 3.58 | 4.30 | 30.40 |
| **3** | **2** | **32** | **2** | 14.00 | 4.00 | 9.25 | 21.10 | 23.75 | 24.90 | 7.10 | 21.20 | 6.95 | 9.80 | 3.98 | 2.35 | 32.10 |
| **3** | **3** | **33** | **2** | 17.00 | 3.50 | 10.10 | 20.10 | 23.85 | 25.20 | 8.10 | 20.60 | 7.29 | 10.15 | 4.52 | 2.15 | 38.70 |
| **4** | **1** | **41** | **2** | 15.50 | 4.50 | 5.20 | 19.00 | 24.20 | 19.10 | 8.35 | 24.85 | 5.20 | 7.35 | 3.90 | 4.10 | 33.80 |
| **4** | **2** | **42** | **2** | 17.00 | 3.80 | 8.90 | 20.20 | 26.15 | 27.25 | 9.10 | 19.70 | 7.25 | 11.55 | 4.36 | 1.65 | 40.30 |
| **4** | **3** | **43** | **2** | 21.00 | 4.30 | 9.70 | 20.60 | 27.20 | 28.20 | 8.65 | 18.55 | 7.75 | 11.80 | 4.65 | 1.75 | 41.40 |
| **1** | **1** | **11** | **3** | 12.50 | 7.35 | 4.20 | 9.95 | 17.45 | 9.20 | 6.10 | 23.38 | 2.60 | 3.90 | 2.12 | 5.25 | 26.10 |
| **1** | **2** | **12** | **3** | 16.25 | 6.50 | 6.10 | 11.45 | 15.85 | 12.25 | 6.80 | 23.40 | 2.49 | 4.10 | 2.10 | 4.10 | 26.40 |
| **1** | **3** | **13** | **3** | 18.00 | 7.10 | 6.90 | 10.75 | 18.30 | 13.90 | 5.10 | 22.70 | 2.70 | 4.60 | 2.35 | 4.45 | 26.90 |
| **2** | **1** | **21** | **3** | 17.50 | 7.70 | 5.90 | 14.15 | 21.10 | 16.80 | 5.30 | 26.12 | 3.59 | 5.90 | 2.86 | 4.10 | 30.05 |
| **2** | **2** | **22** | **3** | 20.00 | 4.50 | 6.75 | 15.10 | 22.40 | 16.90 | 5.90 | 25.93 | 4.15 | 6.20 | 2.85 | 2.95 | 32.00 |
| **2** | **3** | **23** | **3** | 17.00 | 6.00 | 6.50 | 14.35 | 22.85 | 15.80 | 6.85 | 25.55 | 4.75 | 7.65 | 3.29 | 2.85 | 31.00 |
| **3** | **1** | **31** | **3** | 17.00 | 3.50 | 5.30 | 19.60 | 22.50 | 18.10 | 7.25 | 25.58 | 4.28 | 7.45 | 3.25 | 4.10 | 30.40 |
| **3** | **2** | **32** | **3** | 16.00 | 3.65 | 7.75 | 19.25 | 23.85 | 24.50 | 9.15 | 20.90 | 6.55 | 10.20 | 4.23 | 2.15 | 36.50 |
| **3** | **3** | **33** | **3** | 20.00 | 2.10 | 8.85 | 21.50 | 24.90 | 26.85 | 9.30 | 20.30 | 7.10 | 11.20 | 4.16 | 3.15 | 37.30 |
| **4** | **1** | **41** | **3** | 15.75 | 6.00 | 7.20 | 16.70 | 22.35 | 21.30 | 6.50 | 24.82 | 4.95 | 7.65 | 3.75 | 3.35 | 34.20 |
| **4** | **2** | **42** | **3** | 14.00 | 4.70 | 11.85 | 19.10 | 25.70 | 28.10 | 8.25 | 19.38 | 7.54 | 10.95 | 4.68 | 2.20 | 42.10 |
| **4** | **3** | **43** | **3** | 18.00 | 3.60 | 11.45 | 20.30 | 24.90 | 30.25 | 9.65 | 18.80 | 7.80 | 12.65 | 5.25 | 2.00 | 42.90 |
